# A review of Vietnam’s healthcare reform through the Direction of Healthcare Activities (DOHA)

**DOI:** 10.1186/s12199-017-0682-z

**Published:** 2017-10-30

**Authors:** Kyoko Takashima, Koji Wada, Ton Thanh Tra, Derek R. Smith

**Affiliations:** 1 JICA Project for Strengthening Medical Services in Northwest Provinces, Hanoi, Vietnam; 2 Bureau of International Health Cooperation, National Center for Global Health and Medicine, 1-21-1 Toyama, Shinjuku-ku, Tokyo, 162-8655 Japan; 3 Department of Quality Management, Cho Ray Hospital, Ho Chi Minh City, Vietnam; 4 College of Public Health, Medical and Veterinary Sciences, James Cook University, Townsville, Australia

**Keywords:** Direction of Healthcare Activities (DOHA), Health system, Quality, Referral, Vietnam

## Abstract

**Objective:**

This article provides a comprehensive review of the healthcare reform process driven by the Vietnamese Ministry of Health’s *Direction of Healthcare Activities* (DOHA) scheme.

**Methods:**

We reviewed policy documents relating to DOHA, along with historical literature and background information describing its formation.

**Results:**

DOHA (*Chỉ đạo tuyến* in Vietnamese) literally means *guidance line or level* in English. It requires healthcare facilities at higher government administration levels to support those at lower levels (the four levels being central, provincial, district, and commune), to help lower level hospitals to provide medical services for local communities in primary care settings and reduce the number of patients in higher level (central and provincial) hospitals. Since the 1990s, there have been too many patients attending higher level hospitals, and DOHA has therefore focused on technical skills transfer training to help alleviate this situation. Designated core central hospitals now provide technical skills transfer to provincial hospitals. Professional technical lists for each level of health facility have enabled strong commitment and proactive ownership of the process of training management in both higher and lower level hospitals.

**Conclusion:**

The DOHA scheme has accelerated the necessary up-skilling of healthcare at lower level public hospitals across Vietnam. These reforms are highly relevant for other countries with limited healthcare resources.

## Background

Alongside rapid economic development, the health status of people in Vietnam has significantly improved in recent years, with the life expectancy at birth increasing from 71 years in 1990 to 76 years in 2015 [1]. Infant mortality rates (under 5 years of age) decreased from 58 deaths per 1000 live births in 1990 to 18 in 2015 [2]; and the proportion of under-five-year-olds who were underweight decreased from 37% in 1993 to 14% in 2015 [3, 4]. However, wide disparities remain in core health indicators between rural and urban residents, across different regions, and among population groups [4]. Disease patterns in contemporary Vietnam are changing, with the main societal health problems shifting from maternal and child care and infectious diseases to non-communicable diseases and traffic-related injuries [5]. Vietnam also has one of the most rapidly aging populations in the world [6, 7], with an increasing demand for quality healthcare services and new issues likely to emerge in the health sector in future years [4]. The country’s government is now being forced to consider not only a plan for developing healthcare manpower and improving health infrastructure such as facilities and equipment, but also for better management of limited healthcare resources and reforming health financing to improve overall efficiency [4].

The healthcare workforce in Vietnam is currently insufficient to meet manpower norms and practical needs [4], with the number of physicians in 2015 (around eight per 10,000 population) [4] being quite low when compared to other countries in Southeast Asia [8]. Healthcare resources should be appropriately distributed to meet needs [9], but there is currently an imbalanced distribution of human resources and a shortage of manpower in Vietnam, especially of highly specialized physicians in fields such as cancer, palliative care, and mental health [10, 11]. Mountainous and remote areas have severe shortages of healthcare workers [12], with the number of physicians per population in the North West, Central Highland, and Mekong Delta Regions being lower than the national average [4]. Most healthcare workers in remote areas manage with a shortage of medical equipment and training. They have limited opportunities to use advanced diagnosis and treatment methods and maintain and improve their professional ability [4]. The quality of healthcare services is therefore lower in remote areas than more urban regions.

Healthcare facilities in Vietnam are divided into four levels by administrative structure: central (Level I); provincial (Level II), covering a population of 1–2 million; district (Level III), covering 100,000–200,000; and commune (Level IV), covering around 5000–10,000 [13]. This structure is set out in Article 81 of Chapter VIII of the 2009 Law on Examination and Treatment [6], which covers the organizational system of medical examination and treatment establishments. Level I hospitals include central hospitals owned by the Ministry of Health and city hospitals owned by municipalities such as Hanoi or Ho Chi Minh City. Level II, III, and IV hospitals are owned by local provincial governments, such as the people’s committee responsible for allocating finance and human resources. The provincial or district health department is responsible for their professional management under the Vietnamese Ministry of Health.

The healthcare system has a mixture of public and private provision. The number of private hospitals is increasing, but as of 2014, only 6% of all healthcare facilities were privately owned [14]. Private hospitals, however, now provide more than 60% of outpatient services and have become an important component of the national health system [15]. A health insurance system was introduced in 1993, and the government has made a considerable effort to achieve universal coverage, reaching 77% of the population in 2015 [4]. Recently, the government has announced a target of 90% health insurance coverage by 2020 [16]. Reform of the organizational structure of healthcare at all levels is currently underway, as set out in the master plan for Vietnam’s health system development to 2025 [4]. The plan explains that having too many facilities can create instability and inconsistency, especially at the grassroots level. It also leads to a shortage of human resources, increased administrative expenditure, and the decreased effectiveness of health services.

Having too many patients in higher level hospitals has become an urgent problem in recent years, with two to three patients sharing a bed becoming common in many central and provincial hospitals [17]. Bed occupancy rates have reached 120–160%, especially in the central hospitals of some large cities [4]. Overcrowding in higher level healthcare facilities may have several causes, including limited healthcare quality in lower level facilities in districts and communes, and even in provincial hospitals; increasing expectations of service quality; improvement in convenience of transportation from remote areas to central areas; and limited differences in hospital fees at different administrative levels [13]. This may lead to a drain on resources in higher level hospitals and subsequent wastage at lower levels. If the current situation is not improved, this situation will eventually result in major inefficiencies across the entire Vietnamese health care system.

The Ministry of Health in Vietnam has managed healthcare provision through a system known as the *Direction of Healthcare Activities* (DOHA) since 1961 [18]. This system requires health facilities at higher administrative levels to support those at lower levels to enable them to deliver medical services for local communities in primary care settings. The contents of DOHA have been modified and adjusted over time based on the need for medical care, but the word “DOHA” has been retained in the context of medical care reform [18]. DOHA currently focuses on reducing the burden on higher level hospitals, particularly central hospitals, which still have too many patients seeking health care. The healthcare system in Vietnam is not well known outside the country and information regarding DOHA is rarely available in English, and as such, the aim of this article was to review the health reform process through the DOHA scheme in contemporary Vietnam.

## Methods

We reviewed appropriate policy documents setting out the concept of DOHA, the historical background and information regarding the formation of DOHA. One of the authors (KT) is bilingual (Vietnamese and English) and spent several years working on a project to enhance DOHA activities with staff from the Ministry of Health, Vietnam, funded by the *Japan International Cooperation Agency* (JICA). During this project, a number of official documents were translated into English. The JICA project also supported provincial hospitals in the northwestern part of Vietnam to obtain basic data on the number of patients referred from district hospitals and to central hospitals, to monitor the situation.

## Results

### DOHA framework

DOHA (or *Chỉ đạo tuyến* in Vietnamese) literally means *guidance line or level* in English. The term DOHA has been used as the English translation for some time, and documents translated by the Ministry of Health commonly use phrases such as “Technical Direction” or “Giving guidance to hospitals at lower levels,” with further explanations including “regional medical guidance activities” and “guidance and support from higher to lower level hospitals”.

The DOHA has two major missions [18]:

1. To build a sound collaboration network and support system among health facilities, particularly those at higher and lower levels, to help ensure equity of health and deliver quality healthcare services to all Vietnamese people.
2. To address the burden of too many patients in higher level centers. This means supporting improvements in the quality of healthcare services provided at lower levels, particularly training and technical skills transfer activities to improve trust and respond to social demands.

DOHA has recently focused on technology transfer training from central to provincial hospitals to help alleviate the excessive numbers of patients in provincial or district hospitals. So, DOHA covers collaboration activities among hospitals at different levels [18].

### Laws and regulations related to DOHA

The concept behind DOHA first appeared in Vietnam as one of the five tasks for hospitals in 1961 and since then, DOHA has been clarified in hospital regulations in 1969, 1971, and 1978 as an important hospital activity [19]. In 1997, regulations set out that DOHA was one of the seven tasks of hospitals and its organizational structure was also clarified [18]. In 1998, the Minister of Health identified Bach Mai Hospital, the central hospital in northern Vietnam, located in Hanoi, as a pilot hospital for a trial implementation of DOHA [20]. Bach Mai Hospital set up a DOHA department, staffed by two medical doctors, and strengthened its training provision to lower level hospitals [6] [20]. Since then, more practical and detailed regulations for DOHA have been developed. Table 1 shows the six current areas of DOHA based on Decision 4026/QD-BYT in 2010 [18].

**Table 1:** Contents of DOHA based on the Decision 4026/QD-BYT by the Ministry of Health, Vietnam (2010)

Structures	Contents
Supporting lower level hospitals to ensure medical services.	・Survey the current situation at lower levels in terms of material facilities, equipment, human resources, professional capacity, training needs, technical exchange and others, supporting demands from lower levels. ・Build up and organize DOHA activity implementation for lower levels. ・Check the implementation process of professional regulations and the technical progress of lower levels. ・Provide feedback from referred patients, plus timely updates on current technical and professional errors, special diagnosis cases, and lessons learnt. ・Technical assistance given to lower level facilities upon request. ・Coordinate with lower levels to build up a referral system across the assigned area. ・Coordinate with higher levels in the implementation of DOHA activities in the field of medical services.
Training and technical skills transfer for lower level hospitals.	・Organize training and technical skills transfer courses for healthcare workers at lower level healthcare facilities. ・Support healthcare professionals from lower levels to practice and improve their professional skills at higher level facilities. ・Receive training and technical skills transfer support from higher levels.
Scientific research	・Implement scientific research on professional knowledge and management of DOHA activities. ・Coordinate with higher levels and instruct lower levels in implementing scientific research activities.
Support for community health service	・Coordinate with higher levels and instruct lower levels to implement community-oriented activities like primary healthcare services, environment protection, prevention of epidemic diseases, and national healthcare programs. ・Be ready to support lower level hospitals in the event of any disaster or social problems.
Regular meeting and review	・Coordinate higher and lower levels to organize meetings, regular activities to draw out professional lessons, preliminary review, and final review for DOHA activities.
Role of Level I hospitals (central hospitals).	・Assist the Ministry of Health in giving direction for professional knowledge, national professional and specialized network system development plan, and also coordinate with hospitals which have DOHA assignments. ・Build up training courses and implement them to help lower levels to develop their professional techniques and specialties to improve quality of emergency aid, diagnosis, treatment, and prevention. ・Build plans and give direction to healthcare services at lower level facilities to implement national and international programs. ・Check, monitor, and evaluate professional and technical activities of lower level facilities. ・Organize annual review and summary, making regular and ad hoc reports on the results of DOHA activities nationwide to Ministry of Health.

*DOHA* Direction of Healthcare Activities

Central hospitals (Level I) have a DOHA center and training center. These draw on the functional capacity of these healthcare facilities and play a key role in coordination and management of DOHA across specialties at both the central hospitals and in hospitals in the provincial DOHA network. The concept of DOHA has developed to promote the sharing of roles and improved collaboration among healthcare facilities, to improve the quality of medical services.

Table 2 shows the laws and regulations related to DOHA, which have changed over time [18]. DOHA was defined as one of the seven responsibilities of hospitals in the hospital regulation of 1997, the others being medical service, staff training, scientific research, prevention activities, international cooperation, and hospital management. In 2004, according to the “Instructions for strengthening DOHA activities in medical services,” the purpose of DOHA was to demonstrate the fulfillment of medical services for local people and guarantee equitable healthcare. The instructions included the importance of establishment of DOHA networks, covering central, provincial, district, and commune levels, and concrete implementation procedures such as planning and approval processes for DOHA and its budget. In 2009, the Law on Examination and Treatment stated that provision of guidance and support for use of medical technology to lower levels was part of the responsibilities of higher level hospitals [6].

**Table 2:** Laws and regulations related to the *Direction of Healthcare Activities* (DOHA)

Government documents	Year	Reference	Title	Related documents
MOH Decision	1997	1895/QD-BYT	Hospital Regulation	
MOH Instruction	2004	09/CT-BYT	Instructions for strengthening DOHA activities in medical services	
Law	2009	2009/QH12	Law on medical examination and treatment	
MOH Decision	2010	4026/QD-BYT	Assignment of DOHA Activities in medical services	9/QD-BYT, 2004
MOH Decision	2012	5068/QD-BYT	Implementation procedures on training and technical transfer for health service packages under the 1816 project	1816/QD-BYT, 2008 (called 1816 project)
Prime Ministerial Decision	2013	92/QD-TTg	Approval of the scheme on hospital overload reduction (2013–2020)	
MOH Decision	2013	774/QD-BYT	Satellite hospital project (2013–2020)	
MOH Circular	2013	43/TT-BYT	Professional technical lists for each level of health facilities	
Prime Minister Decision	2013	14/QD-TTg	Implementation plan of term-limited rotation for medical practitioners in medical facilities	
MOH Circular	2014	14/TT-BYT	Regulation on referral among health facilities	

*MOH* Ministry of Health

The Vietnamese Minister of Health also signed Decision No. 1816/QD-BYT, in 2008, on the project “Dispatching healthcare workers from higher-level hospitals to support lower-level hospitals with the aim of improving quality of examination and treatment” [18]. By the end of 2009, one and a half years after its initial implementation, an average reduction of 30% in overcrowding at higher level facilities had been observed [18]. However, bypassing lower level facilities is still relatively common and many people still seek care at provincial and central hospitals to treat diseases that could easily be handled at the district or even commune level [18]. This bypassing leads to an increased burden on high-level facilities and under-utilization of services at lower levels, causing unnecessary waste across the whole system. This project was further supported, and staff rotation strengthened, by Decision No. 5068/QD-BYT (Table 2) in 2012, which regulates the content of some training and technical skills transfer as well as supporting the Satellite Hospital Project [21].

### Project for hospital overload reduction (2013–2020)

In 2013, the Vietnamese Prime Minister set out targets to be achieved by the year 2020. The target for the bed occupancy rate of central hospitals in Hanoi and Ho Chi Minh City (which exceeded 120%) was set at 100% or less; and district and provincial hospitals were expected to achieve occupancy rates of 80% [22]. There was also a limit set for the number of patients per day seen by each doctor, to reduce patient waiting times. This decision indicates a commitment to address patient numbers comprehensively. There have been a number of projects to support this, including improvements in hospital facilities to increase the number of beds, introduction of family doctors, strengthening of primary healthcare and preventive medicine, and improved health education and promotion activities for local people. DOHA-related policy documents published in 2013 based on this prime ministerial decision were designed to further improve the feasibility of technical transfer activities under DOHA. Technical skills transfer training under DOHA is based on a standard list of medical technologies that can be implemented at each level in the Ministry of Health circular on “Professional technical lists for each level of health facilities (43/TT-BYT)” [23]. The list consists of 28 clinical fields with 17,216 medical technologies and specifies medical technologies required at each level [23]. Lower level hospitals that have received technology transfer training from higher level hospitals must submit documents such as training completion reports to the provincial Department of Health to obtain official approval and a license to introduce the new technologies or procedures. These activities call for strong commitment and proactive ownership of the process of training management from both higher and lower level hospitals.

### Satellite Hospital Project (2013–2020)

The Satellite Hospital Project (2013–2020) evolved from the Vietnamese Prime Minister’s target to reduce the number of patients in higher level hospitals [21]. This project aims to upgrade the technical capacity for examination and treatment in provincial hospitals designated as satellite hospitals through technical skills transfer by central or core hospitals. This project is expected to reduce the number of patients referred from provincial to central hospitals and alleviate the subsequent overload at central hospitals. Satellite and core hospitals are required to set out a development plan covering facilities, medical equipment, and human resources. The first phase of the project covered 2013 to 2015, and the second phase from 2016 to 2020. Five specialties with very high bed occupancy rates were prioritized in the first phase: oncology, traumatology, cardiology, obstetrics, and pediatrics. During 2016–2020, more specialties will be added, including endocrinology, neurology, intensive care, hematology, and infection control [24].

The main elements of the project are:

1. To formulate and develop a network of satellite hospitals. The first phase, from 2013 to 2015, prioritized investment in 48 provincial hospitals as satellites of 14 core hospitals (eight owned by the Ministry of Health and six under the Department of Health Service of Ho Chi Minh City);
2. To enhance the examination and treatment capacity of satellite hospitals via training, technical skills transfer, and tele-consultations using information technology (telemedicine);
3. To invest in core and satellite hospitals to improve manpower, facilities, medical equipment, referral means, and information technology to ensure effective and sustainable transfer of techniques. Satellite hospitals are expected to perform and maintain the new techniques by themselves. They are also expected not to refer patients to core hospitals for these techniques except where they have insufficient capacity.

### Referral of patients based on DOHA

In Vietnam, the term “referral” means patient referred from lower to higher level healthcare facilities [22]. Referral activity is part of DOHA, and therefore the referral process is managed by hospitals’ DOHA departments. Patient referral is a process to manage the issue that healthcare staff at lower levels often have insufficient resources (facilities, equipment, and diagnosis and treatment capacity) to manage patients’ clinical conditions. The Ministry of Health defines referral activities as any activities including “patient referral activity and management of patient referral information among health facilities.” This is also covered by the Ministry of Health Circular 14/TT-BYT in 2014 on regulation on referral among health facilities [18]. Patient referral among healthcare facilities takes into account the technical capacity of each healthcare facility, based on the standard lists of medical technologies.

One of the expected outcomes is reducing the number of patients referred to higher level hospitals, together with improved outcomes of treatment at all levels. Some provincial hospitals have collected the number of patients referred from district hospitals and to central hospitals. Table 3 shows the trends in Yen Bai provincial hospital, about 150 km to the northwest of Hanoi, the capital city of Vietnam, serving a population of 771,000 [18]. The number of patients referred from Yen Bai provincial hospital to higher level hospitals in Hanoi steadily increased from 2010 to 2012. After the implementation of the strengthened DOHA activities from 2013, the level of referrals remained stable. Slightly fewer patients were examined in the provincial hospital during 2013.

**Table 3:** Trend in patient examinations and transfers at Yen Bai provincial hospital (2010–2015)*

	2010	2011	2012	2013	2014	2015
Number of patients examined	123,691	102,404	91,262	84,557	101,424	102,011
Number of patients referred from Yen Bai provincial hospital to the central hospital in Hanoi						
Outpatients	2171	2692	3132	3665	3821	3404
Inpatients	629	626	804	941	904	1046

*Adapted from Reference [18]

## Discussion

We reviewed the health reform process through DOHA in Vietnam. Within the framework of DOHA, there have been various activities and regulatory interventions over the past 50 years. To continue the improvement of the quality of care in hospitals, higher level of hospitals could take initiatives for technical transfer to lower level hospitals via the regulatory framework.

There have also been additional advantages of DOHA. It has, for example, improved the relationships between higher and lower level hospitals by promoting mutual understanding among staff. This will facilitate communication about patients and help to avoid unnecessary transfers. Collaboration among different level hospitals is part of DOHA’s mission [18]. DOHA also encouraged district and provincial hospitals to look at their own hospital services more critically and start thinking about how to improve the health service and provide more patient-centered care, as well as focusing on investment in applying new medical technologies themselves, through DOHA’s technical transfer program [18].

A limitation of DOHA has been difficulties in identifying the impact of DOHA activities. Various other factors, such as economic and infrastructural development, may have led to an increase in demand for sophisticated medical care and subsequent improvements in access to higher level hospitals. Despite DOHA’s effort, it may be difficult to expect an immediate reduction in the number of patients being referred to higher level hospitals, as shown in Table 3. However, monitoring the number of patients being referred to higher level hospitals will be necessary to help plan which areas of clinical training should be undertaken through DOHA.

Although DOHA includes technical transfer training for medical doctors, training for managers and other healthcare providers should also be expanded. It was previously considered that nursing practice is simple enough for each provincial hospital to improve the quality of nursing by themselves. However, in order to deliver high-quality patient-centered care, all health professionals should be educated as members of an interdisciplinary team with professional communication and team collaboration. Training programs in patient safety, infection control, and nursing management (issues which are relatively recent in Vietnam) have now been conducted through DOHA and have included nurses and other health care workers. In the future, DOHA is expected to place more emphasis on these issues and provide greater opportunities to share good practice in Vietnamese healthcare.

## Conclusion

DOHA is the system in Vietnam requiring healthcare facilities at higher administrative levels to support their lower level counterparts to improve the quality of healthcare services provided to all Vietnamese citizens. Since the 1990s, there have been too many patients using higher level hospitals, and DOHA has therefore focused on technical skills transfer training. The DOHA scheme has accelerated the necessary up-scaling of medical technologies across the country from higher to lower administrative levels. This system is highly applicable to other countries with limited healthcare resources wishing to improve the quality of healthcare services.

## Abbreviations

DOHA: Direction of Healthcare Activities
MOH: Ministry of Health
